# Miniature Spectroscopes with Two-Dimensional Guided-Mode Resonant Metal Grating Filters Integrated on a Photodiode Array

**DOI:** 10.3390/ma11101924

**Published:** 2018-10-10

**Authors:** Yoshiaki Kanamori, Daisuke Ema, Kazuhiro Hane

**Affiliations:** Department of Finemechanics, Tohoku University, Miyagi 980-8579, Japan; ron@hane.mech.tohoku.ac.jp (D.E.); hane2@hane.mech.tohoku.ac.jp (K.H.)

**Keywords:** spectroscopes, metamaterial, plasmonics, structural color filters, photodiodes

## Abstract

A small spectroscope with 25 color sensors was fabricated by combining metamaterial color filters and Si photodiodes. The metamaterial color filters consisted of guided-mode resonant metal gratings with subwavelength two-dimensional periodic structures. Transmittance characteristics of the color filters were designed to obtain peak wavelengths proportional to grating periods. For each color sensor, a peak wavelength of the spectral sensitivity could be tuned in the range of visible wavelengths by adjusting each grating period. By performing spectrum reconstruction using Tikhonov regularization, the spectrum of an incident light was obtained from the signal of photodiodes. Several monochromatic lights were made incident on the fabricated device and the spectral characteristics of the incident light were reconstructed from the output signals obtained from the respective color sensors. The peak wavelengths of the reconstructed spectra were in good agreement with the center wavelengths of the monochromatic lights.

## 1. Introduction

Color filters, which work as wavelength selective filters, have been used in image sensors and liquid crystal displays. In recent years, since the discovery of extraordinary transmission phenomenon based on surface plasmon by Ebbesen et al. in 1998 [1], plasmonic color filters using metal nanostructures have been actively studied [2,3,4,5,6]. Plasmonic color filters have various advantages over the conventional color filters using pigment, such that various color characteristics can be realized depending on the structural shapes with thicknesses of just tens of nanometers. Therefore, plasmonic color filters for many colors can be fabricated on the same substrate by a single fabrication process, unlike the conventional color filters using pigment. Also, plasmonic color filters have high compatibility with complementary metal–oxide–semiconductor and charge-coupled devices based on semiconductor microfabrication technologies, compared with color filters using conventional pigments. Furthermore, in recent years, a nanoimprint technology in which submicron structures are easily formed using molds has been advanced [7,8,9,10,11,12,13,14,15,16,17,18], and improved productivity of plasmonic nanostructures can be expected.

Spectroscopes are widely used [19] as instruments for measuring the energy intensity for each wavelength of light. Since most spectroscopes use diffraction gratings as wavelength selective elements, a certain propagation distance is required to separate diffracted waves spatially. Therefore, it is difficult to miniaturize the spectroscopes that use diffraction gratings. Moreover, precise optical axis adjustment is required for assembling the numerous optical components in the construction of the spectroscopes, which results in high cost. In industrial fields, colorimeters using spectroscopes are used as devices for measuring color. To meet the increasing demand for spectroscopic devices, such as the color management of products and food quality control, cost reduction and the downsizing of spectroscopic devices are strongly required.

In recent years, novel spectroscopes fabricated by combining plasmonic color filter array and photodiode array have been reported [20,21,22,23,24,25,26]. A color filter is formed on each photodiode, and spectral information is calculated using output signals obtained from photodiodes. Such filter array spectroscopes do not require long propagation distances like the ones using diffraction gratings and are thought to have the possibility of expanding the range of use of spectroscopic devices as ultrasmall and inexpensive spectroscopes. However, the wavelength selectivity of color filters used for the filter array spectroscopes reported are insufficient. Instead, increasing the number of filters and implementation of an improved calculation algorithm have been attempted to improve the spectroscopic characteristics of devices [22]. Increasing the number of filters leads to an increase in the light receiving area, contrary to miniaturization. Also, complicated calculation processing leads to the load and delay of calculation processing. Therefore, to improve filter array spectroscope devices, the improvement of the filter characteristics itself is required. Although guided-mode resonant gratings are known as high-efficiency wavelength selective filters [27,28,29,30,31,32,33,34,35,36], they function as reflection type wavelength selective filters. Transmission type wavelength selective filters are necessary for application in filter array spectroscopes.

As mentioned above, various plasmonic color filters have been reported so far. However, as far as we know, there are only few reports on filter array spectroscopes integrating color filters and photodiodes. Therefore, research on the improvement of their characteristics is required. In this study, we design and fabricate a filter array spectroscope and evaluate the characteristics of the fabricated spectroscopic device integrating newly designed color filters and photodiode array. There are no reports of filter array spectroscopes combining guided-mode resonant gratings and photodiodes, as far as we know. We have newly designed transmission type wavelength selective filters based on guided-mode resonant metal gratings with subwavelength two-dimensional (2D) periodic structures without polarization dependency for normal incident light.

## 2. Device Configuration

**Figure 1:**
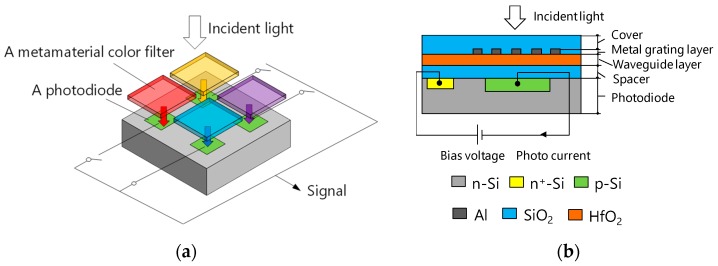
Schematics of proposed devices. (**a**) Perspective view in the case of 2 × 2 color sensors; (**b**) cross-sectional view of one particular color sensor.

Figure 1a shows a conceptual diagram of the proposed spectroscopic device. A plurality of color sensors with different spectral sensitivity characteristics, in which metamaterial color filters having different spectral characteristics are stacked and arranged on each photodiode, are arranged. Incident wavelengths selected by the color filters enter the photodiodes, the output signals of the photodiodes are sequentially read out, and the spectral characteristics of the incident light are obtained by calculation processing. Figure 1b shows the cross-sectional view of a color sensor consisting of a metamaterial color filter formed on an Si photodiode through a spacer layer. The metamaterial color filter consists of a guided-mode resonant metal grating covered with a SiO_2_ layer.

The guided-mode resonant metal grating consists of a 2D Al grating layer formed on an HfO_2_ guided layer. The guided layer is formed on the SiO_2_ spacer layer. Since the effective refractive index of the guided layer is higher than that of the surroundings, the guided layer functions as a planar waveguide and strongly confines the resonant wavelength. Transmitted light generates only zeroth order diffraction because the grating period is smaller than the resonant wavelength. The grating groove is filled with SiO_2_. Incident light is impinged from the SiO_2_ cover layer. Photocurrent generated in the photodiode is read out as an output signal. By changing the period of the metal grating layer of each color filter, it is possible to control the resonant wavelength and to extract spectral information from each photodiode.

## 3. Design and Numerical Analysis

### 3.1. Design of Color Filters

**Figure 2:**
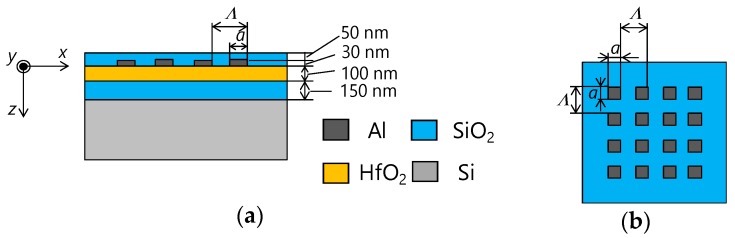
Calculation model of one color filter element. (**a**) Cross-sectional view; (**b**) top view.

Numerical analysis was carried out using rigorous coupled-wave analysis (RCWA), which yields accurate results using Maxwell’s equations in the frequency domain [37,38]. Figure 2 shows a calculation model of one color filter element. The electric field, magnetic field, and propagation direction of incident light are parallel to the *x*, *y*, and *z* axes, respectively. A 50-nm-thick SiO_2_ cover layer, 30-nm-thick 2D-periodic Al nanodot array, 100-nm-thick HfO_2_ waveguide layer, and a 150-nm-thick SiO_2_ spacer are formed on an Si substrate of photodiode. Here, *Λ* and *a* are the grating period and nanodot size, respectively. The color filters have polarization independent because of 2D subwavelength gratings with the same periods for the *x* and *y* directions.

**Figure 3:**
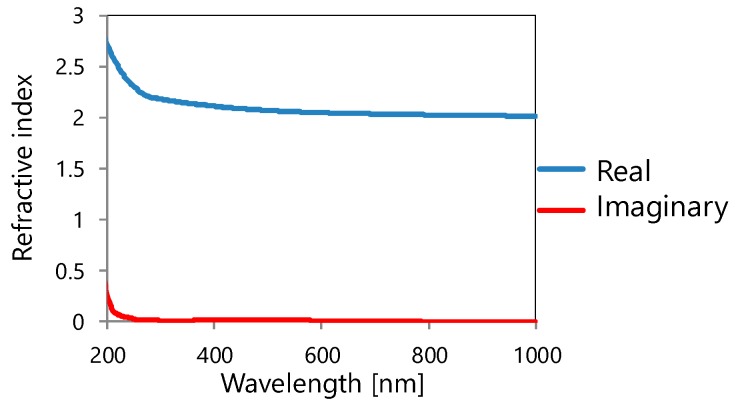
Refractive index of HfO_2_ used for calculation.

Normally, incident light from outside was made to pass through the cover layer, and transmittance spectrum at the Si side of the SiO_2_/Si interface was calculated to obtain spectral characteristics of the device. For optical designing of the structure integrated with the color filter and photodiode, spectral characteristics, including the influence of reflection and interference by the Si substrate, were calculated instead of just obtaining the transmission spectrum of only the color filter. The incident light polarized along the *x*-axis was normally incident and the total transmittance obtained by combining all the transmitted diffracted waves was calculated. Refractive indices of Al, Si, and SiO_2_ in references [39,40,41], respectively, were used for the calculation. Figure 3 shows real and imaginary parts of the refractive index of HfO_2_ used for the calculation, which was measured by a spectroscopic ellipsometer.

**Figure 4:**
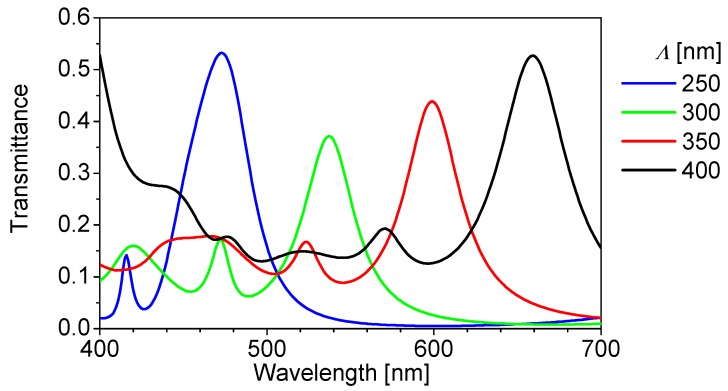
Calculated transmittance spectra penetrating a Si substrate for several grating periods. *a*/*Λ* is fixed to be 0.8.

Figure 4 shows the calculated transmittance spectra penetrating a Si substrate for several grating periods. Here, *a*/*Λ* was fixed to be 0.8. Figure 4 illustrates that the peak wavelength of the transmission spectrum can be controlled mainly through the grating period. The peak wavelength is shifted to the longer wavelength side as the period increases, which agrees with the principle of guided-mode resonant gratings. From this result, we could design the structures that exhibit functions of spectroscopes, including a reflection and interference effect, due to the influence of the interface between the SiO_2_ space and Si photodiode. The output signal actually obtained by the color sensor is outputted as a characteristic signal, which is the product of the transmission spectrum shown in Figure 4 and the spectral sensitivity characteristic of the Si photodiode.

**Figure 5:**
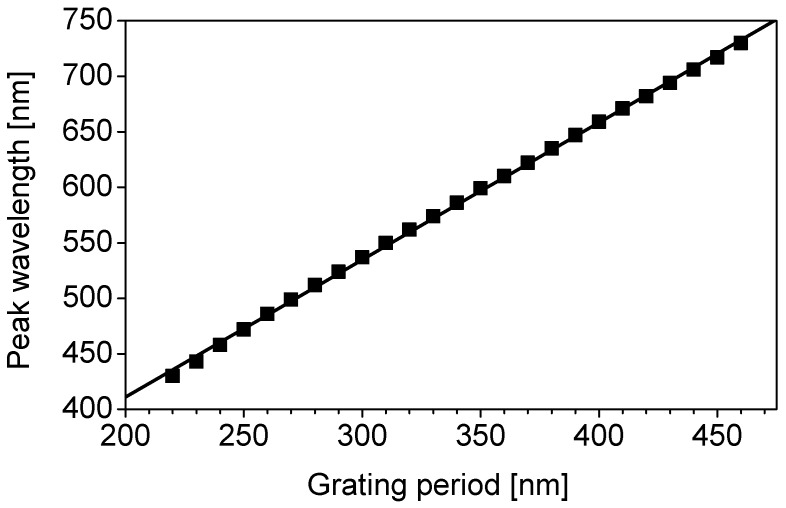
Peak wavelength as a function of grating period, extracted from the results of Figure 4.

Figure 5 shows the peak wavelength as a function of the grating period extracted from the results of Figure 4. It can be seen that the peak wavelength shifts in proportion to the grating period in the entire visible wavelength range. The linear fitting equation for Figure 5 is given by the following equation:

(1) $$\lambda_{\mathrm{peak}}=1.24\times \Lambda +163.4$$

Here, *λ*_peak_ is a peak wavelength.

### 3.2. Spectrum Reconstruction: Principle and Calculation Examples

An output signal from each color sensor is the integrated amount of the energy spectrum received by each photodiode and it cannot be decomposed into spectral components. Therefore, in order to reconstruct the spectral characteristic of the input light from the output signal, calculation between output signals is required. To solve the inverse problem of determining the input signals (spectrum of incident light) from the output signals, spectral characteristics are calculated using the Tikhonov regularization method [42]. Consider a spectroscopic device composed of *b* color sensors. *b* is the number of color sensors. The photocurrent of each color sensor is obtained as a product of the spectral characteristic of incident light and the wavelength sensitivity characteristic of each color sensor, integrated over the wavelength. The photocurrent is expressed in matrix format as follows:

(2) O=SI

Here, **O** (A) is a 1 × *b* column vector of photocurrent. **S** (A/W) is a *b* × *c* matrix of wavelength sensitivity, and **I** (W) is a 1 × *c* column vector of spectral characteristics of incident light. *c* is a wavelength division number. **O** and **S** are measured experimentally, and **I** is to be determined. In order to solve this inverse problem, **I** is calculated using the matrix, **M**, obtained by the Tikhonov regularization method, as shown below:

(3) I=MO 

**M** is a *c* × *b* matrix. Besides, because the spectral characteristic cannot be a negative value, the following condition is added:

(4) I=I (I>0) or 0 (I≤0)

Equations (3) and (4) are solved to obtain the spectral characteristics.

Next, several incident light spectra were designed (designed spectra) and compared with the incident light spectra (reconstructed spectra) calculated using Equations (3) and (4). It should be noted that *b* was set to 25 and the calculated wavelength range to be integrated was 400 to 700 nm. Twenty-five color filters with different grating periods between 210 and 450 nm at intervals of 10 nm were used for the matrix, **S**. Some of the 25 filter characteristics are shown in Figure 4. Here, the sensitivity of the Si photodiode is ignored for simplicity.

**Figure 6:**
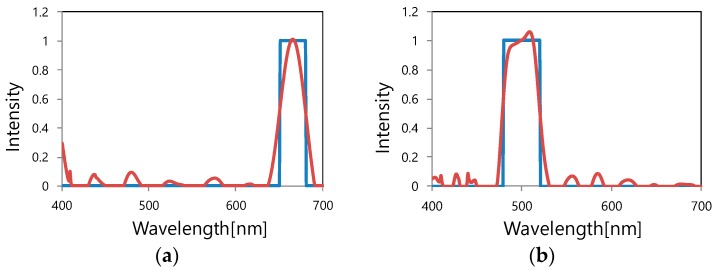

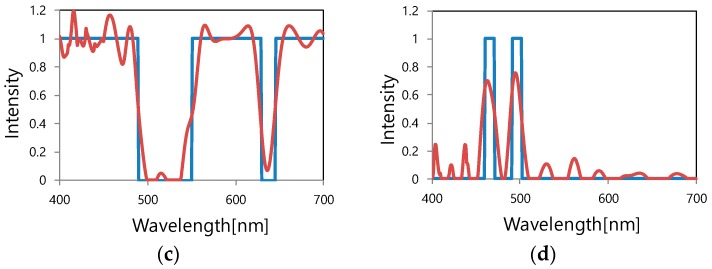
Simulation of spectral reconstruction (Blue line: Designed spectrum; red line: Reconstructed spectrum). (**a**) Single band spectrum near 700 nm in wavelength; (**b**) single band spectrum at a center wavelength of 500 nm; (**c**) multiple band-stop spectrum; (**d**) multiple peak spectrum.

The calculation results are shown in Figure 6. As shown in Figure 6a,b, the peak positions of the designed spectrum and the reconstructed spectrum coincide with each other, and the bandwidths also substantially coincide. However, the sharp edges of the designed spectrum take rounded shapes like the Gaussian distribution in the reconstructed spectrum. This can be attributed to the coarse resolution because just 25 filters are provided across a wavelength range of 300 nm. It can be improved by increasing the number of filters. Moreover, as can be seen from Figure 6c,d, it is possible to calculate the results for multiple peaks. Figure 6d reveals that as the width of the designed spectrum becomes narrower, the peak position of reconstructed data is in good agreement with the designed peak position, but the peak intensity of the reconstructed spectrum decreases. These results suggest that optical spectra in a wavelength range of 400 to 700 nm can be obtained by using the proposed devices; however, there is room for improvement.

## 4. Fabrication

**Figure 7:**
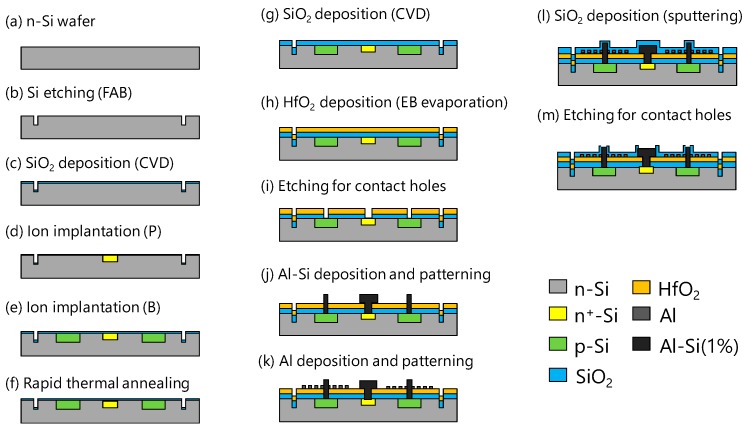
Process steps.

Figure 7 shows the process steps. An n-type Si substrate of a 400 μm thickness is etched by a fast atom beam (FAB) to form alignment marks (Figure 7a,b). Next, to prevent metal contamination, an SiO_2_ protective film with a thickness of 5 nm is formed by chemical vapor deposition (CVD) (Figure 7c). Then, P ions are implanted into the contact area of electrodes to form n+ -Si (Figure 7d). After that, ion implantation of B is performed to form p-Si (Figure 7e). Besides, rapid thermal annealing is performed to activate ions simultaneously with the recovery of crystal from damage due to ion implanted ions (Figure 7f). Next, as a spacer, SiO_2_ is formed by CVD (Figure 7g). Then, as a waveguide layer, HfO_2_ is deposited by electron-beam (EB) evaporation (Figure 7h).

After that, SiO_2_ and HfO_2_ etching is performed to make contact holes (Figure 7i). Next, the Al-Si (1%) layer with a thickness of 400 nm is formed by sputtering, and wet etching is performed to form electrodes (Figure 7j). After Al film formation by sputtering, nanodot array structures are patterned by EB lithography, followed by FAB etching (Figure 7k). Next, an SiO_2_ cover layer is formed by sputtering (Figure 7l). Finally, SiO_2_ in the electrode pad portion is etched (Figure 7m).

**Figure 8:**
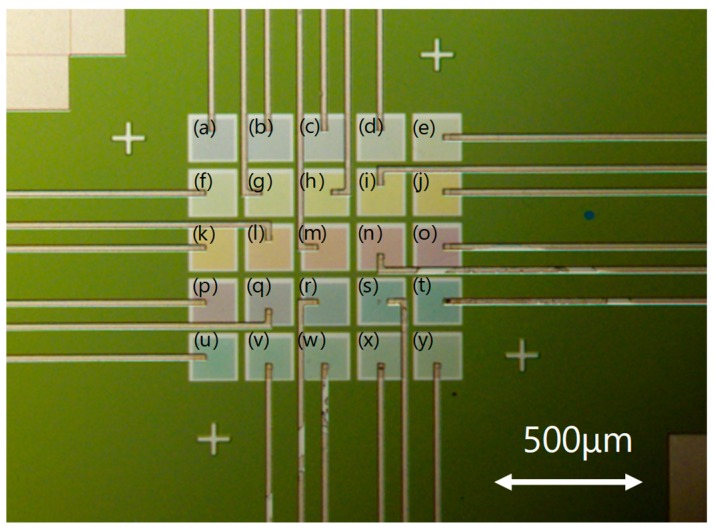
Optical microphotographs of fabricated color filters on the photodiode array. (**a**) *Λ* = 220 nm, (**b**) *Λ* = 230 nm, (**c**) *Λ* = 240 nm, (**d**) *Λ* = 250 nm, (**e**) *Λ* = 260 nm, (**f**) *Λ* = 270 nm, (**g**) *Λ* = 280 nm, (**h**) *Λ* = 290 nm, (**i**) *Λ* = 300 nm, (**j**) *Λ* = 310 nm, (**k**) *Λ* = 320 nm, (**l**) *Λ* = 330 nm, (**m**) *Λ* = 340 nm, (**n**) *Λ* = 350 nm, (**o**) *Λ* = 360 nm, (**p**) *Λ* = 370 nm, (**q**) *Λ* = 380 nm, (**r**) *Λ* = 390 nm, (**s**) *Λ* = 400 nm, (**t**) *Λ* = 410 nm, (**u**) *Λ* = 420 nm, (**v**) *Λ* = 430 nm, (**w**) *Λ* = 440 nm, (**x**) *Λ* = 450 nm, and (**y**) *Λ* = 460 nm.

Figure 8 shows a reflection image of an optical microscope of color filters fabricated on a photodiode array. The filters are fabricated in 25 patterns, with grating periods between 220 and 460 nm at increments of 10 nm, which correspond (a) to (y) in the figure. All filters are 150 μm × 150 μm in size. Although the color filter is designed as a transmissive type, the structural color, which depends on the period of the structure, can also be confirmed in the reflected images. The wiring pattern connected from each photodiode can also be confirmed.

**Figure 9:**
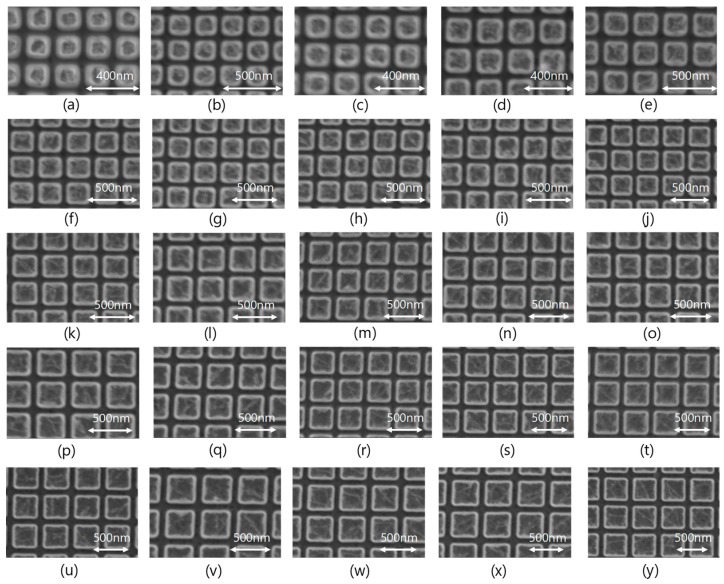
SEM images of fabricated color filters. (**a**) *Λ* = 220 nm, (**b**) *Λ* = 230 nm, (**c**) *Λ* = 240 nm, (**d**) *Λ* = 250 nm, (**e**) *Λ* = 260 nm, (**f**) *Λ* = 270 nm, (**g**) *Λ* = 280 nm, (**h**) *Λ* = 290 nm, (**i**) *Λ* = 300 nm, (**j**) *Λ* = 310 nm, (**k**) *Λ* = 320 nm, (**l**) *Λ* = 330 nm, (**m**) *Λ* = 340 nm, (**n**) *Λ* = 350 nm, (**o**) *Λ* = 360 nm, (**p**) *Λ* = 370 nm, (**q**) *Λ* = 380 nm, (**r**) *Λ* = 390 nm, (**s**) *Λ* = 400 nm, (**t**) *Λ* = 410 nm, (**u**) *Λ* = 420 nm, (**v**) *Λ* = 430 nm, (**w**) *Λ* = 440 nm, (**x**) *Λ* = 450 nm, and (**y**) *Λ* = 460 nm.

Figure 9 shows scanning electron microscope (SEM) images of the fabricated color filters corresponding to the symbols (a) to (y) of Figure 8. All filters are designed to be 0.8 of the *a*/*Λ* ratio. It can be seen that all the filters are fabricated accurately.

## 5. Measured Results and Discussion

**Figure 10:**
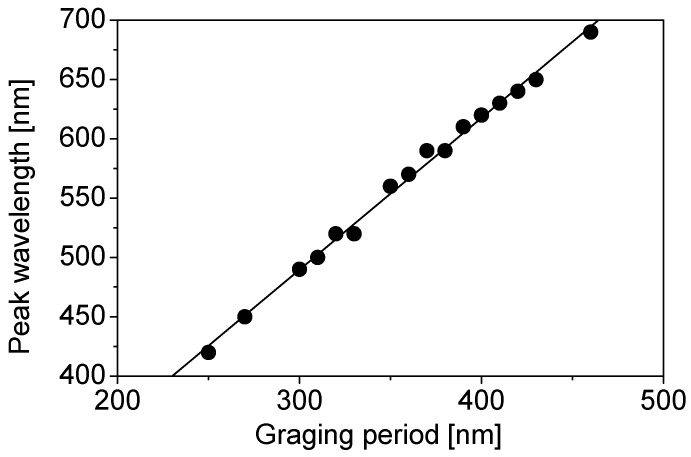
Dependence of the peak wavelength of the spectral sensitivity on the grating period.

Spectral sensitivity characteristics of color sensors were measured using a broadband spectral response measurement system (CEP-25BXS, Bunkou Keiki Co., Ltd., Tokyo, Japan). In this measurement, the wavelength resolution was set to 10 nm. Different color sensors exhibited different spectral sensitivity characteristics. Figure 10 shows the relationship between the grating period and the peak wavelength of the spectral sensitivity. The peak wavelength is linearly dependent on the grating period, and the slope of the straight line almost agrees with that of the calculated peak wavelength shift of the color filters shown in Figure 5. The linear fitting equation for Figure 10 is given by the following equation:

(5) $$\lambda_{\mathrm{peak}}=1.28\times \Lambda +105.4$$

**Figure 11:**
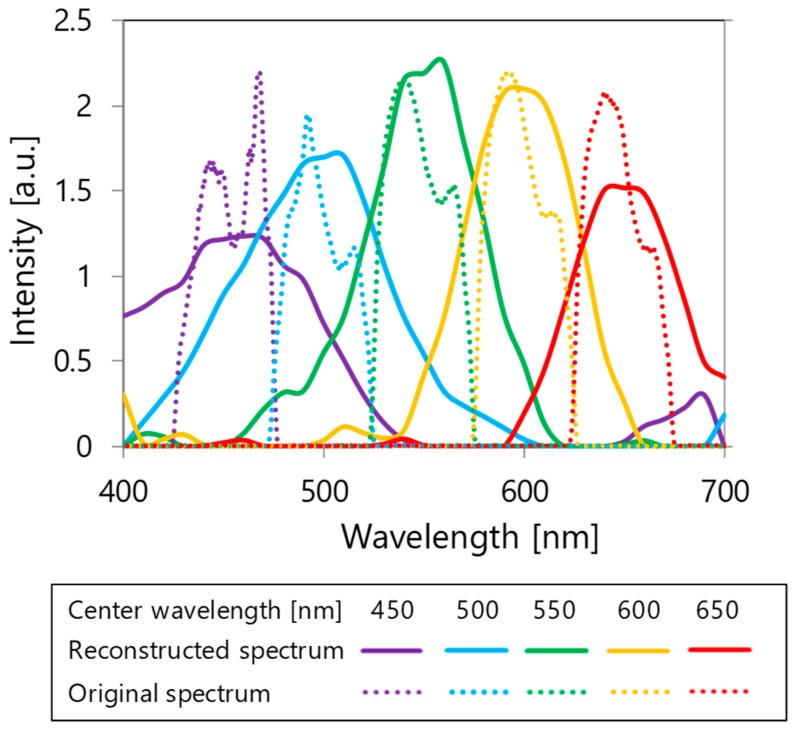
Spectral reconstruction.

When the monochromatic light with wavelengths of 450, 500, 550, 600, and 650 nm is incident, the spectral characteristics of the incident light are reconstructed from the output signals obtained by the respective color sensors. Spectral characteristics of the reconstructed incident light are shown with solid lines in Figure 11. For comparison, spectral characteristics of the incident light measured with a commercially available spectrometer (HR4000CG-UV-NIR, Ocean optics, Inc., Largo, FL, USA) are shown as original spectra with dotted lines. The peak wavelength of the reconstructed spectrum can be confirmed to be near that of the original spectrum. Therefore, we believe that the spectroscopic measurement was successfully performed using the fabricated device. However, reconstructed spectra become broader compared to the original spectra. This increment in width can be attributed to the coarse resolution because only 25 filters are provided across a wavelength range of 300 nm. The reconstructed spectra can be improved by increasing the number of filters.

**Figure 12:**
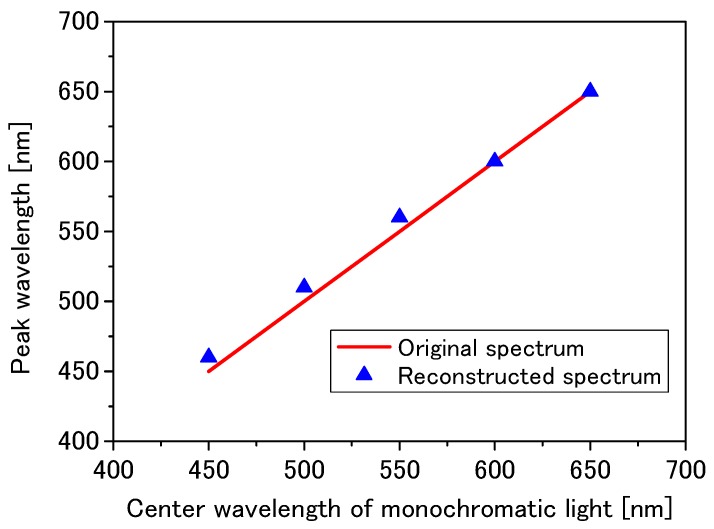
Dependence of the peak wavelengths of reconstructed spectra on the center wavelength of the incident monochromatic lights.

Figure 12 shows the relationship between the peak wavelengths of reconstructed spectra and the center wavelength of monochromatic lights extracted from the results of Figure 11. It is found that the peak wavelengths of the reconstructed spectra are in good agreement with the center wavelengths of the monochromatic lights.

## 6. Conclusions

We fabricated a filter array spectroscope consisting of metamaterial color filters and Si photodiodes. The color filters based on guided-mode resonant metal gratings were newly designed. The peak wavelength of the transmission spectrum could be controlled mainly by the grating period. The filters were fabricated in 25 patterns, with grating periods between 220 and 460 nm at increments of 10 nm. SEM images of fabricated color filters confirmed that all the filters were fabricated accurately. The peak wavelength of the spectral sensitivity was found to be linearly dependent on the grating period. Several monochromatic lights were made incident on the fabricated device and the spectral characteristics of the incident light were reconstructed from the output signals obtained from the respective color sensors. The peak wavelengths of the reconstructed spectra were in good agreement with the center wavelengths of the monochromatic lights.
